# Selective versus comprehensive emergency management in Korea

**DOI:** 10.1186/2193-1801-3-602

**Published:** 2014-10-15

**Authors:** Kyoo-Man Ha, Hyeon-Mun Oh

**Affiliations:** Department of Emergency Management, Inje University, 197 Inje-ro, Gimhae, 621-749 Korea; Department of Regional Infrastructure Engineering, Kangwon National University, 1 Kangwondaehak-gil, Chuncheon, 200-701 Korea

**Keywords:** Government, Industry, Community, Household, Culture

## Abstract

In spite of Korean governments’ efforts, many emergency management practitioners wonder whether what is actually being practiced is selective or comprehensive management. Using a qualitative content analysis and experiences in practice, the article analyzes the barriers to selective emergency management and the paths to comprehensive emergency management via the same three management elements: stakeholders, phases of the emergency management lifecycle, and hazards and impacts. Four analytical levels are considered: central government level, industry level, community level, and household level. Korea, despite its self-praise, has to transform its selective emergency management into comprehensive emergency management in time.

## Introduction

Officials of the South Korean (hereinafter Korean) government have recently and frequently boasted that Korea has made considerable progress in implementing comprehensive emergency management. They have officially proclaimed that Korea has improved many aspects of comprehensive emergency management, such as all stakeholders, the four phases of the emergency management lifecycle, and all hazards and related impacts, at every level. However, many emergency management practitioners wonder whether what is actually being practiced is selective or comprehensive management, considering that there is room for improvement in terms of comprehensive emergency management. For example, the ferry Sewol sank on April 16, 2014 and then no individual or institution reacted to the initial phase of disaster response. Further, only 172 out of 476 passengers were rescued and thus the nation argues the necessity of setting up the national emergency operation framework (MOSPA, 2014). Therefore, it is necessary to study, as a major research question, whether or not Korea has really progressed in comprehensive emergency management.

This study aims to substantially facilitate the adoption of comprehensive emergency management in Korea by examining barriers to the current selective emergency management and suggest ways to achieve comprehensive emergency management. Both qualitative content analysis and practice experiences are utilized as major methodologies to collect information and data on the two types of management. In so doing, this article systematically compares the two emergency management types via the same three major elements, namely, stakeholders, phases of the emergency management lifecycle, and hazards and impacts, at four analytical units: central government level, industry level, community level, and household level. This article will emphasize that Korea must transform its selective emergency management to comprehensive emergency management in time, as a major tenet.

### Theoretical overview

#### Concept comparison between selective and comprehensive emergency management

Two kinds of emergency management are discussed in this article: selective emergency management and comprehensive emergency management. They are opposite concepts in terms of the entire direction of emergency management. Selective emergency management, which is newly named here, is a traditional type, whereas comprehensive emergency management, which is being widely practiced, is a modern type (FEMA, 2010b: 2.5–2.6; Zessin, 2006: 8–9). An increasing number of organizations have tried to utilize comprehensive emergency management, but many still rely on selective emergency management under their own limited environments.

The term ‘selective’ means having the power or function of selecting. Selective emergency management means that its components are not comprehensive but selective. Among so many emergency issues, selective emergency management tends to apply for the participation of selective stakeholders in the process of emergency management, unequal support for selective phases of emergency management lifecycle, and inclusion of selective hazards and impacts into emergency management. In other words, selective emergency management comes to happen for the goal of managing not all issues but some imminent issues in the field, because of bounded personnel, resources, and strategies. However, it is also true that the some cases of emergency management strategically rely on selective emergency management, though they have abundant personnel and resources. Related groups wrongfully believe that selective emergency management is more effective for the goal of emergency management than other measures.

Because selective emergency management does not play a role in reliably predicting or preventing a new emergency, it provides poor management. Nevertheless, selective emergency management has its own benefits. For example, it decreases financial costs. When the comprehensive aspect of emergency management is not depended on, selective emergency management significantly cuts financial costs (Conrad, Patton, Parikshak, and Kralovich, 2003: 268–272). However, such savings are not oriented for long-term but for short-term benefit.

Some nations have heavily utilized selective emergency management in the international community, according to a literature review. More accurately, they have not had much choice but to depend on selective emergency management under their own critical emergency environments. For example, government has played almost every role in managing emergencies in Nigeria, particularly without the support of its business corporations, voluntary organizations, and other entities. As a result, oil pipeline disasters have frequently occurred in the country. Without the support of other stakeholders, government alone could not properly prevent oil pipeline disasters (Onuoha, 2009: 384–386).

In India, poor coordination between government institutions and non-governmental organizations (NGO) led to serious corruption of relief operations, government’s misuse of funds, and inefficiency of emergency distribution system in 1999. In short, the lack of NGO activities led to selective emergency management (Thomalla and Schmuck, 2004: 380–381). In Iran, the nursing training program was selective, considering that nurses failed to update their information and skills periodically. Without an updated training program, which is the phase of emergency mitigation, nurses committed more mistakes in real situations in 2003 (Nasrabadi, Naji, Mirzabeigi, and Dadbakhs, 2007: 15–16).

Some hospital programs in the United States were based on selective emergency management, specifically through their emergency planning, and as a result such programs could not achieve their targeted goals (Drezner, Courson, Roberts, Mosesso, Link, and Maron, 2007: 148–154). In addition, because emergency psychology programs in some areas lacked a systematic set of helping actions, emergency responders failed to recover from post-traumatic distress. Such programs should have included a broader spectrum of related needs and problems (Ruzek, Brymer, Jacobs, Layne, Vernberg, and Watson, 2007: 30–33). The above two cases show that the United States also had a selective problem with the phases of emergency preparedness/recovery.

On the other hand, the term ‘comprehensive’ means involving or covering a lot of area. Comprehensive emergency management therefore means that its components are comprehensive, that is, comprehensive emergency management supports the importance of all stakeholders participating in emergency management, equally supports all phases of the emergency management lifecycle, and covers all hazards and impacts in emergency management, contrary to what happens in selective emergency management (FEMA. 2010a: 1.8–1.9; Laidlaw, Spennemann, and Allan, 2008: 71–75).

Comprehensive emergency management does not deal with only one side of emergency management. Rather, it deals with every side of emergency management, including not only the demand side but also the supply side (Erickson, Champion, Klein, Ross, Neal, and Snyder, 2008: 2254–2261). By the same token, comprehensive emergency management should deal with both internal emergencies and external emergencies. It deals not only with routine emergencies but also with non-routine emergencies (Capri, Ignaccolo, and Inturri, 2009: 89–94; Reif, Liffers, Norrester, and Peal, 2010: 34–37).

Comprehensive emergency management is a sort of holistic model, especially when considering that this type of management plays a role in guiding public policy and research directions. Through comprehensive emergency management, emergency planning has been practiced and at the same time related researches have been organized (Adams, 2008: 26; Dickson, 1992: 82–85). Many nations have recently made efforts to adopt comprehensive emergency management. As a noteworthy case, the United States came to nationally institutionalize comprehensive emergency management when it set up the Federal Emergency Management Agency (FEMA) in 1979, during the Carter Administration (FEMA. 2010a: 1.8).

According to a review of the literature, the effectiveness of comprehensive emergency management has been proved in many countries, as in the aspect of sustainable emergency planning during earthquakes in Iran in the last 20 years (Hosseini, Jafari, Hosseini, Mansouri, and Hosseinioon, 2009: 661–663) and the comprehensive researches after the mine disaster in Spain in 1998 (Guerrero, Lozano, and Rueda-Cantuche, 2008: 24–36). In Sri Lanka, comprehensive emergency management focused on burying human bodies in dignified places or in dignified ways as well as on identifying the missing or the dead, particularly with the help of damage control exercise. In these instances, not only legal troubles were dealt with but psychological interventions were improved as well (Sumathipala, Siribaddana, and Perera, 2006: 252–256).

#### Framework for turning from selective to comprehensive emergency management in Korea

**Figure 1:**
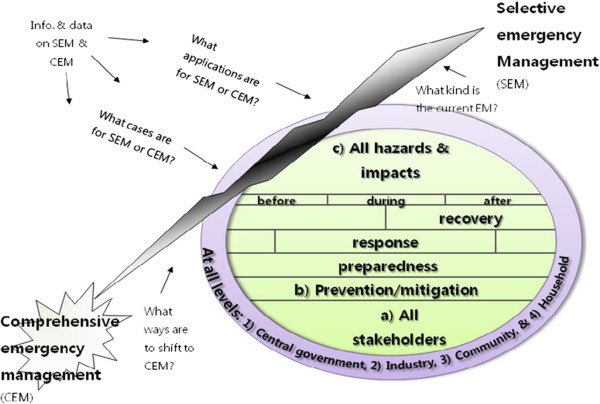
**Analytical framework.** Sources: FEMA, 2009: 2–2; NEMA, 2014.

This article relies heavily on a qualitative content analysis as a methodology. However, because real practices are equally important in the field of emergency management, this article tries to reflect what emergency personnel on the emergency site have felt. In other words, this study collected and interpreted related information and data on selective and comprehensive emergency management via both qualitative content analysis and practice experiences, as shown in Figure 1.

To compare selective emergency management and comprehensive emergency management in Korea, this study asked four major questions. First, what kinds of emergency management has Korea recently practiced? Second, what applications has Korea utilized for either selective or comprehensive emergency management? Third, what significant cases has Korea produced for either selective or comprehensive emergency management at each analytical level? Fourth, is there any way for Korea to shift to comprehensive emergency management from its current situation?

Some practitioners insist that selective emergency management would still be needed for Korea, because of limits in the related environment, as in the shortage of emergency funds, the lack of emergency personnel, and limits on other materials and capabilities (Choi, 2010). However, mainly because comprehensive emergency management is a sort of emergency management principle, which should always be applied in the field of emergency management (FEMA. 2010b: 2.5-2.6), it is necessary for Korea to definitely change any aspect of selective emergency management to the complex of comprehensive emergency management.

In the meantime, very few researchers have studied how to improve selective emergency management or how to embody comprehensive emergency management for Korea, even though diverse international scholars have focused on examining these two management types for their developed nations or some developed nations. In addition, almost no systematic study has been conducted to compare selective and comprehensive emergency management for Korea (Lee and Cho, 2010: A92-A93; Marietta, 2012: 110–122). Thus, this article works on the topic as a pioneering study.

This article will maintain that selective emergency management should be promptly changed to comprehensive emergency management in Korea. In particular, comprehensive emergency management has to be practiced at all levels in the near future. To systematically compare the two kinds of emergency management, the same three major components of both types of management, namely, a) stakeholders, b) the phases of the emergency management lifecycle, and c) hazards and impacts, will be examined at all four analytical levels: 1) central government level, 2) industry level, 3) community level, and 4) household level. These four levels were chosen because they make up the major parts of emergency management levels, thus showing the whole direction toward which both selective and comprehensive emergency management should focus.

### Barriers to selective emergency management in Korea

#### Central government level

Officials of the National Emergency Management Agency (NEMA) have played dominant roles in deciding important issues on emergency management at the central government level in Korea. As a note, the NEMA will be officially turned into the National Safety Administration (NSA, which is tentatively known) by incorporating with the National Maritime Policy sooner or later. Because the majority of NEMA personnel consist of firefighters and civil engineers, they, as public servants, have taken part in deciding emergency management. Further, parties outside NEMA, such as representatives from business corporations, voluntary organizations, and the community, among others, have not been allowed to participate in the decision making of NEMA (NEMA, 2014).

An analysis of government documents shows that NEMA has generally recognized the importance of the four phases of the emergency management lifecycle. However, NEMA has realistically focused on supporting the phases of response and recovery far more than the other phases. For example, firefighting emphasizes the phase of response, whereas civil engineering advocates the phase of recovery. Although some officials have recently advocated support for the new phase of protection, the major policy of NEMA continues to support the phases of response and recovery (Kang, 2008: 1099–1101).

NEMA is formally considered a single national agency that deals with all kinds of hazards and their related impacts. However, the institution has substantially supported firefighting and response to floods due to typhoon. Because NEMA personnel comprise both firefighters and civil engineers, it is no wonder that these two kinds of hazards and impacts have been constantly vindicated. Moreover, it is true that these two hazards and impacts are frequent in Korea.

Based on the above-mentioned facts, it is hardly possible to maintain that the central government has adopted comprehensive emergency management. Although NEMA officials have officially proclaimed the goal of dealing with all kinds of hazards and their related impacts with equal support for the four phases of the emergency management lifecycle, the detailed policy of NEMA shows that it has increasingly moved toward the aspects of selective emergency management. Further, one of the biggest barriers is that both firefighters and civil engineers have always formed the backbone of decision making and, therefore, preferred emergency management policy.

#### Industry level

Although business leaders in conglomerates such as Samsung, Hyundai, LG, Doosan, SK, Kia, and Lotte, among others, have recognized the importance of business continuity planning (BCP) for their assets via their export requirement, they have allowed only top managers to decide the important issues in emergency management. A similar pattern has been found in the case of small and medium-sized corporations as a culture, though not many of them know the meaning of BCP. In short, low-ranking stakeholders have been excluded from the related decision-making process. Rather, such stakeholders have purely worked for emergency teams.

Many corporations have tried to instinctively support the phases of prevention/mitigation and recovery to increase their business benefits. In other words, they are willing to prevent, mitigate, or recover from emergencies around their business sites, without systematically recognizing the importance of the four phases of the emergency management lifecycle. This means that they are unwilling to equally advocate the phases of preparedness and response, based on their own cost-benefit analysis. In particular, many business leaders in small and medium-sized corporations are not aware of the four phases for their emergency management.

Regardless of corporation size, the majority of business corporations have tried to set up computer backup systems for their BCP, although accomplishment of this task not been complete. They know that the computer backup system will play a vital role in maintaining their corporate benefits even under conditions of emergency. In short, many corporations do not practice modern BCP but the traditional BCP (Kim, 2009b: 135–138). Besides, not much has been done in terms of dealing with other hazards and related impacts, such as industrial espionage, arson, and earthquake.

Although conglomerates have tried to practice their own BCP, the majority of small and medium-sized corporations have not consolidated their emergency management. Moreover, conglomerate corporations have shown many defects in their BCP, such as only the top managers being involved in decision making in BCP, unequal support for the phases of preparedness and response, and ignoring the danger of other hazards and impacts. Therefore, based on the above-mentioned facts, it is estimated that industry in Korea has relied heavily on the use of selective emergency management.

#### Community level

The community consists of the local government, community-based organizations (CBO), and voluntary organizations, to name a few. More stakeholders have been allowed to take a part in deciding emergency management issues at the community level than at the central government level, mainly because the heads of the community want to be reelected based on their experience and ability in deciding on emergency management issues. Specifically, representatives from voluntary organizations and CBOs have been frequently invited to take part in the decision making of the local government. Furthermore, almost all stakeholders have had no choice but to participate during an emergency at the scene of the emergency.

The community has traditionally made efforts to support the phases of response and recovery as a culture, similar to the situation at the central government level. In one sense, because the community deals directly with emergencies on the spot, it has to respond to real emergencies. The community continues to advocate for the phase of recovery, considering that the community longs to get back to its normal life through the recovery phase. In another sense, because the central government supports the phases of response and recovery, local governments, as a sub-government or as a key member of the community, have followed the same pattern, with the support of voluntary organizations and CBOs.

Local governments include fire stations, the sections of Bangjae, and police stations. These three are supposed to deal with all hazards and impacts in the community: fire stations fight fires and their impacts, sections of Bangjae deal with other hazards and their impacts, and police stations deal with terror and its impacts. Nonetheless, local governments with the support of voluntary organizations and CBOs have currently come forward to deal with two hazards and their impacts, namely, fires and floods caused by typhoons. The sections of Bangjae literally mean to work for disaster prevention against all hazards and impacts, but substantially have worked for civil engineering management against floods caused by typhoon. In addition, because police stations lack manpower, they have not been effective in fighting terror in the community.

With the above-mentioned facts in mind, it is very certain that the community depends upon selective emergency management. The community has set up a series of emergency operation plans (EOP) and then used them in particular situations instead of utilizing one comprehensive EOP. When a fire occurs, the community uses its own EOP on fire. If floods break out because of typhoon, the community relies on the EOP regarding floods accompanied by typhoon. When drought hits, the community uses the third or the fourth EOP. In short, the community’s establishment of many EOPs to address each hazard and impact is a typical case of selective emergency management (McCann, 2009: 331–334).

#### Household level

In general, almost all members of a family are willing to participate in dealing with incoming emergency as well as in making decisions on important emergency issues. Because the value of filial duty as children has been more importantly appreciated than that of loyalty to the nation in Korea, the relationship among family members has traditionally been very strong as a culture, particularly in emergency management. It is also true that each household has well reflected what the others outside the family have worried about in decision making.

In some aspects, without the appropriate guidance of the government, many households do not have a good understanding of the importance of the four phases of the emergency management lifecycle. Rather, they have relied on their instincts to respond to and recover from emergency, even without recognizing the scientific sequence of the four phases. However, an increasing number of households have been informed about the four phases, and they have subsequently tried to include them in their emergency management. In so doing, the household’s support for the phases of prevention/mitigation and preparedness has increased, although incrementally.

Many households understand how to deal with fires and flooding accompanying a typhoon, because these two have often happened around them. However, they are at a loss when dealing with other hazards and their related impact (Kim, 2009a: 222–226). Thus, when either avian flu or foot and mouth disease broke out at the start of the twenty-first century, some residents in rural areas returned home without being sanitized after visiting the emergency area. Therefore, the diseases came to spread quickly and widely.

Based on these analyses, it is not possible to evaluate efforts that households have made to use comprehensive emergency management, although it is exceptionally true that all stakeholders have taken part in both decision making and the first line of defense against emergency. Households have not played a noteworthy role at all in equally supporting the four phases of the emergency management lifecycle and in dealing with all hazards and the related impact. Thus, it is safe to conclude that households have depended upon selective emergency management.

### Paths to comprehensive emergency management in Korea

Korea is currently stuck with selective emergency management, as shown in Table 1. On the basis of the previous section on “Barriers to selective emergency management in Korea,” this evaluation has been double checked with interviews of 16 emergency management practitioners, including four government officials, four business experts, four community leaders, and four members of households. Each of the four groups replied not only to its own level but also to the other three levels, for objectivity. In short, interview results have been directly or indirectly reflected to the simplified evaluation.

**Table 1:** **Simplified evaluation on reality of current emergency management**

All levels/three management components	a) All stakeholders	b) Four phases of emergency management lifecycle	c) All hazards and impacts
1) Central government level	○	◑	◑
2) Industry level	○	◑	○
3) Community level	◑	◑	○
4) Household level	●	○	○

Note: ○ = selective emergency management, ◑ = selective + comprehensive emergency management, and ● = comprehensive emergency management.

### Central government level

Because the guidelines of NEMA have adopted selective emergency management, it is inevitable for NEMA to, in substance, change its policy to comprehensive emergency management. Considering that the central government has traditionally dominated the public and private relationship in Korea, it is NEMA that should initiate correcting its biggest barrier to the principle of emergency management. In particular, NEMA should consider changing the composition of its human resources by radically reforming its personnel administration.

In a sense, firefighters and civil engineers, as public servants, have tried to reflect what other stakeholders outside NEMA have worried about regarding NEMA’s decision-making process on emergency management by holding seminars, conferences, and a complaints window via the Internet. However, nobody outside NEMA has ever been allowed to directly take part in deciding on important issues within NEMA. In addition, considering the constant proportion of firefighters and civil engineers among NEMA personnel, NEMA has not sincerely listened to what other stakeholders want. Therefore, NEMA should immediately allow other stakeholders to participate in its decision-making process.

NEMA must equally support the phases of prevention/mitigation and preparedness as well as the phases of response and recovery. From a short-term perspective, it would look better for NEMA to unequivocally advocate for the phases of response and recovery, because of its limited resources and capabilities. From a long-term perspective, however, it is definitely appropriate for NEMA to fairly support the phases of prevention/mitigation and preparedness to achieve the ultimate goal of emergency management (Bae, 2010: 34–37). The phase of prevention/mitigation will be improved by supporting inspection, legalization, public parks in potential areas, and so on, even as the phase of preparedness will be improved by elaborating the systematic aspect of emergency training.

When other hazards and impacts happen, related catastrophe will swoop down Korea, causing terrible havoc. Mainly because NEMA has not seriously dealt with the danger of other hazards and impacts except for fire and flooding caused by typhoons, the potential of human loss and economic damage may be imminent. Such was the case when drought hit Gangwon province at the beginning of 2009: the economic damage was much heavier than what many had expected. By changing the composition of human resources in NEMA, the central government should include other hazards and impacts into its policy domain, especially in a long-term point of view. New disasters may then be significantly mitigated, unlike the situation at present.

### Industry level

Industry should realize why it has to rely on comprehensive emergency management, particularly when it is based on scientific cost-benefit analysis. When industry invests in comprehensive emergency management, the expected benefits during emergency will outweigh the costs of investment before the emergency. When industry hesitates to invest, the potential cost will be much more than what it has to invest. Therefore, both conglomerates and small and medium-sized corporations must shift from the current selective emergency management to a comprehensive one.

Top-bottom decision making does not work effectively when dealing with a real emergency, because the stakeholders at the bottom are the ones who deal directly with emergencies at the location. Thus, stakeholders at the bottom as well as at the top have to be simultaneously allowed to participate in the decision-making process in business corporations. This may be improved primarily by the top managers’ changing response to who will be the decision makers for BCP. In other words, top managers should realize the importance of not some but all stakeholders for BCP.

Comprehensive emergency management includes well-designed plans and continuity of emergency preparedness (Broz, Levin, Mucha, Pelzei, Wong, Persky, and Hershow, 2009: 1499–1500; Deyle, Chapin, and Baker, 2008: 350–353). Therefore, business corporations should carefully plan BCP and then equally support the phases of preparedness and response, as well as those of prevention/mitigation and recovery. At the same time, they have to continue to support the four phases of the emergency management lifecycle. Without continuously investing in the phases of preparedness and response, the existing efforts toward the phases of prevention/mitigation and recovery will not be smoothly achieved.

Business corporations should include not only a computer backup system but also methods to deal with industrial espionage, arson, earthquake, and others emergencies in their BCP. To comprehensively manage emergencies, industry must take some actions against industrial espionage and arson, which frequently occur in modern society. Moreover, when an earthquake hits, it is easy to guess what will happen to many corporation buildings, which were not built based upon construction codes. A strong earthquake may result in their corporate assets disappearing within a blink of an eye.

### Community level

Many ways may be available for a community to change from selective to comprehensive emergency management. Among such choices, one of the most intriguing ones is the community setting up a single EOP instead of relying on a series of EOPs and using different EOPs for the respective emergency. The community has to include all kinds of emergencies into a single comprehensive EOP after developing a new EOP framework.

A positive aspect is that every stakeholder in a community has successfully participated in fighting emergencies on the spot. It is also good to recognize that many representatives from NGOs have been allowed to take part in the local government’s decision-making process. This is really an exceptional case, compared with the case of the central government. However, the community should allow not many but all stakeholders to participate in the local government’s decision making in the future (Kim, 2008: 12–14; Wiseman, Williamson, and Fritze, 2010: 138–142). In addition, this issue should not be decided by local politics but by the goal of emergency management.

It is necessary for the community to equally support not only the phases of response and recovery but also those of prevention/mitigation and preparedness for comprehensive emergency management. As long as the central government unfairly advocates the phases of response and recovery, it will not be easy for local governments under its influence to attempt to more strongly support the phases of prevention/mitigation and preparedness. However, representatives from NGOs may facilitate a change in this pattern by officially and equally advocating the four phases during the local government’s decision-making process.

The community has to include other hazards and impacts, such as animal diseases, special events, domestic violence, earthquakes, and so on, as well as fires and floods caused by typhoons into a single comprehensive EOP. If done so, particularly with the support of the central government or a national initiative, human loss and economic damage will decrease in the community. In addition, this matter may be solved by local political leaders by proposing the importance of dealing with all hazards and impacts for the entire community (Park, 2009:107–115). Of course, it will be helpful for their reelection, although they assert that it is to deviate from the direction of central government.

### Household level

Households are the most fundamental units in the field of emergency management. Without improving their approach to selective emergency management, the goal of comprehensive emergency management will not be achieved. By reinforcing every aspect of comprehensive emergency management, households will play a role in expanding comprehensive emergency management into the society via a bottom-top approach.

The participation of stakeholders inside and outside the family is positive at the household level. However, the participation of all stakeholders should not be the end; rather it should be a starting point. Based on all stakeholders’ participation, each household needs to professionally utilize emergency psychology programs. Because such programs have been unpopular in Korea, each household has to know how to fully utilize them. For example, a family should take good care of its members who have been victimized, by systematically adopting psychology programs (Dorji, 2006: 544–546).

It is necessary for households to study the sequence of the four phases of the emergency management lifecycle as well as their importance. They can do it in particular via the Internet, because almost all households have access to it. The most vulnerable people in emergencies, such as children or the elderly, have to ask guidance from their family members on how to use the Internet. In addition, those family members who have already studied a bit of comprehensive emergency management need to play an active role in educating their kin regarding the four phases of the emergency management lifecycle (Hwang, Park, Ryu, Lee, Hwang, and Paik, 1999: 177–179). They should pull the trigger to spread advanced information among their households.

Households must realize how to deal with other hazards and related impacts in addition to fires, flood, typhoon, and their impacts. As the members of family become mixed geographically via globalization, some new or foreign diseases such as zoonosis have occurred in households in Korea. Hence, households should pay attention to comprehensively battle swine flu, avian flu, foot and mouth disease, and other animal diseases, as well as other natural disasters such earthquake, drought, and heavy snowstorm.

## Conclusion

This article has tried to show that Korea relies heavily upon selective emergency management, for the purpose of suggesting ways to achieve comprehensive emergency management. In so doing, this article compares selective and comprehensive management via three management elements, namely, stakeholders, the phases of the emergency management lifecycle, and hazards and impacts, at four analytical units: central government level, industry level, community level, and household level. The key finding of the study is that Korea should try to shift from selective to comprehensive emergency management.

One of the most significant contributions of this study is that it provides the framework on how comprehensive emergency management will operate in Korea. Although many public officials or scholars have claimed to support comprehensive emergency management in Korea, almost nobody has ever substantially succeeded in outlining its whole picture. Based on related barriers and paths, this study will play a role in facilitating the real aspects of comprehensive emergency management in the field of Korean emergency management.

One of goals that Korea has recently tried to achieve is to embody comprehensive emergency management in many respects. Therefore, it is necessary for related researchers to study how to improve each aspect of comprehensive emergency management to the fullest extent as soon as possible. This attempt will include at least 12 kinds of researches, multiplying the above-mentioned three management elements and four analytical units, similar to the classification of Table 1 in terms of comprehensive emergency management. As a precondition, scholars have to realize how awkwardly selective emergency management has evolved in Korea.

## Authors’ informations

Kyoo-Man Ha completed his Ph.D. in the Dept. of Political Science in University of Nebraska-Lincoln. He is a researcher for the Research Institute of Radiological Emergency Management, Inje University, Korea. Also, he as an adjunct professor is working for the Dept. of Emergency Management at Inje University. At the same time, he, as Certified Emergency Manager, is serving as the Korean representative for the International Association of Emergency Managers. His research interests include emergency management.

Hyeon-Mun Oh is studying his master degree program in the Department of Regional Infrastructure Engineering, Kangwon National University, Korea. Also, he as Fundamentals of Engineering is working for the Audit and Inspection Division in the Ministry of Security and Public Administration by nationally inspecting civil engineering, architecture, and emergency facilities in 243 local governments under 17 cities and provinces. His research interests include emergency management and civil engineering.
